# *Cylindrospermopsis raciborskii*: review of the distribution, phylogeography, and ecophysiology of a global invasive species

**DOI:** 10.3389/fmicb.2015.00473

**Published:** 2015-05-18

**Authors:** Jorge T. Antunes, Pedro N. Leão, Vítor M. Vasconcelos

**Affiliations:** ^1^Faculty of Sciences, University of Porto, Porto, Portugal; ^2^Interdisciplinary Centre of Marine and Environmental Research (CIIMAR/CIMAR), University of Porto, Porto, Portugal

**Keywords:** allelopathy, climate change, *Cylindrospermopsis raciborskii*, ecophysiology, invasive species, phylogeography

## Abstract

*Cylindrospermopsis raciborskii* is a cyanobacterial species extensively studied for its toxicity, bloom formation and invasiveness potential, which have consequences to public and environmental health. Its current geographical distribution, spanning different climates, suggests that *C. raciborskii* has acquired the status of a cosmopolitan species. From phylogeography studies, a tropical origin for this species seems convincing, with different conjectural routes of expansion toward temperate climates. This expansion may be a result of the species physiological plasticity, or of the existence of different ecotypes with distinct environmental requirements. In particular, *C. raciborskii* is known to tolerate wide temperature and light regimes and presents diverse nutritional strategies. This cyanobacterium is also thought to have benefited from climate change conditions, regarding its invasiveness into temperate climates. Other factors, recently put forward, such as allelopathy, may also be important to its expansion. The effect of *C. raciborskii* in the invaded communities is still mostly unknown but may strongly disturb species diversity at different trophic levels. In this review we present an up-to-date account of the distribution, phylogeography, ecophysiology, as well some preliminary reports of the impact of *C. raciborskii* in different organisms.

## Introduction

The cyanobacterial species *Cylindrospermopsis raciborskii* has gained considerable attention due to its toxicity, bloom formation capacity, and invasiveness into temperate climates. Information about the ecology and distribution of this species was most recently reviewed by Padisák (1997), and a substantial amount of work on this subject has been conducted since. The present review provides a current view of the distribution, phylogeography, and ecophysiology of *C. raciborskii*. In the beginning of this century, the occurrence of this species was reported on an increasing number of countries in both hemispheres, expanding from the original tropical presence. Padisák (1997) has first put forward a proposal for *C. raciborskii* dispersal, supported on ecological and physiological data. Several genetic studies were since conducted, to clarify the phylogeography of this species (Neilan et al., 2003; Gugger et al., 2005; Haande et al., 2008; Moreira et al., 2011a, 2015; Cirés et al., 2014).

In this review we discuss the two proposals that have been advanced to explain the success of this species in different environments. One emphasizes a high phenotypic plasticity of the whole species (Bonilla et al., 2012), while the other underscores the existence of different ecotypes with great intra-specific variability (Chonudomkul et al., 2004; Piccini et al., 2011). We focus our analysis on temperature and light requirements (Briand et al., 2004) as well nutrient dynamics (Istvánovics et al., 2000; Moisander et al., 2012), and climate change (Wiedner et al., 2007; Sinha et al., 2012) because these parameters have been considered to be determinant to *C. raciborskii* invasive success. Other putatively important factors for the invasive behavior, such as allelopathy (Figueredo et al., 2007) are also discussed. Preliminary information regarding the impact of *C. raciborskii* in different ecological groups and on the biodiversity of invaded habitats is also presented. *C. raciborskii* has become a well-studied species due to the production of the toxin cylindrospermopsin (CYN; Ohtani et al., 1992), while later it was shown to produce also paralytic shellfish poisoning (PSP) toxins (Lagos et al., 1999). *C. raciborskii* toxic strains are currently not considered to be broadly distributed, but we argue that monitoring is crucial to obtain a clear picture of the prevalence of toxic strains. The evaluation of the factors that underlay *C. raciborskii* invasiveness will assist in predicting the expansion of *C. raciborskii*, which, due to the toxicity of this species, assumes greater relevance.

## Distribution

**FIGURE 1:**
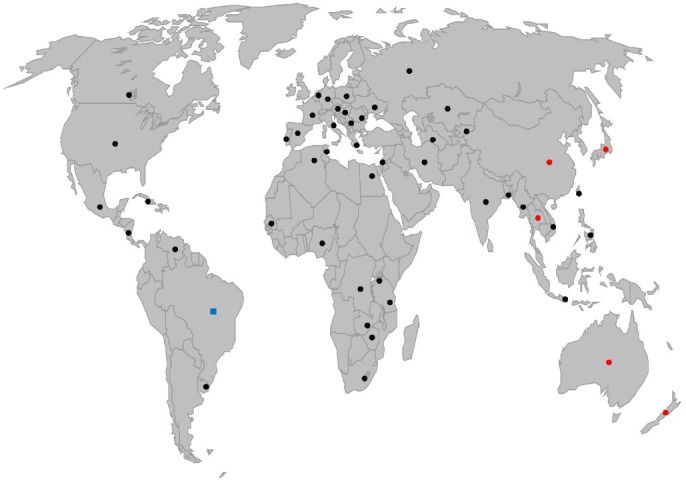
**World map representing countries where *C. raciborskii* presence was reported with cylindrospermopsin or analogs (•) and PSP toxins (▪) producing strains**.

*Cylindrospermopsis raciborskii* was first observed in the island of Java, Indonesia in 1899–1900 and identified by Woloszynska (1912). *C. raciborskii* was later described in India in 1939 (Singh, 1962), and other tropical regions, and consequently considered a tropical species. An increasing number of reports have placed this species in tropical, subtropical and temperate climates, in all continents except Antarctica. The first report of *C. raciborskii* in Europe was in Lake Kastoria, Greece (Skuja, 1937) and it was only again described in Europe in the 1970s (Padisák, 1997). It is postulated that the species colonized from Greece and Hungary toward higher latitudes, near the end of the twentieth century (Padisák, 1997). Currently *C. raciborskii* is distributed throughout most of Europe (Dokulil and Mayer, 1996; Krienitz and Hegewald, 1996; Couté et al., 1997; Borics et al., 2000; Briand et al., 2002; Fastner et al., 2003; Saker et al., 2003). The first description in the African continent probably dates from the end of the nineteenth century (Huber-Pestalozzi, 1938), which may precede Java Island as the first description of the species. In America it was first reported in 1955, in KS, USA (Prescott and Andrews, 1955), in 1979 in Australia (Hawkins et al., 1985), and in the Middle East it was firstly described in one Israeli lake in 1998 (Zohary, 2004; Alster et al., 2010). To this date, the presence of *C. raciborskii* has been reported in an increasing number of countries around the globe, both in the Northern and Southern hemisphere (Figure 1), in rivers, shallow water bodies, lakes and reservoirs. Consequently, the status of this species as tropical or pan-tropical species is disputed, and due to its global distribution, it should probably be considered a cosmopolitan species. Regarding the distribution of toxic strains, *C. raciborskii* was firstly implicated in the “Palm Island Mystery Disease,” in QLD, Australia in 1979 (Hawkins et al., 1985). This incident caused symptoms of gastroenteritis to inhabitants after the water supply was dosed with copper sulfate to control a dense cyanobacterial bloom. Epidemiological studies demonstrated the presence of an unreported species in Australia, *C. raciborskii* (Griffiths and Saker, 2003). Subsequent studies revealed that the *C. raciborskii* cultured isolates from those waters were highly toxic (Hawkins et al., 1985) and the compound it produced was characterized as CYN, a highly potent hepatoxin (Ohtani et al., 1992).

Cylindrospermopsin is a cyclic guanidine-containing alkaloid; for comprehensive reviews about this toxin see Griffiths and Saker (2003), Kinnear (2010), and de la Cruz et al. (2013). As a consequence of CYN production, *C. raciborskii* attained the status of a severe harmful species and not simply a nuisance species. *C. raciborskii* CYN producing strains have, to date, been found only in Australia (Hawkins et al., 1985; Ohtani et al., 1992), New Zealand (Wood and Stirling, 2003), and in East and Southeast Asia (Li et al., 2001a; Chonudomkul et al., 2004; Jiang et al., 2014; Lei et al., 2014; Wimmer et al., 2014; Figure 1). Australian and New Zealand strains produce the CYN analog deoxy-CYN (Norris et al., 1999; Wood and Stirling, 2003; Figure 1), and recently a Thai strain demonstrated the production of two new CYN analogs with probable toxicity (Wimmer et al., 2014). CYN was associated with systems containing *Cylindrospermopsis* in North America (Burns, 2008) and Italy (Messineo et al., 2010), however no North American, European nor Middle Eastern strain has been found yet either to produce CYN or to contain the CYN biosynthesis genes (Neilan et al., 2003; Kellmann et al., 2006; Yilmaz et al., 2008; Alster et al., 2010). The fact that CYN producing strains have been identified in a limited set of countries does not exclude an eventual geographical expansion of toxic strains. CYN is known to be produced by other cyanobacterial species: *Umezakia natans* (Harada et al., 1994), *Aphanizomenon ovalisporum* (Banker et al., 1997; Shaw et al., 1999), *Anabaena bergii* (Schembri et al., 2001), *Raphidiopsis curvata* (Li et al., 2001b), and *Anabaena lapponica* (Spoof et al., 2006) and *Aphanizomenon flos-aquae* (Preußel et al., 2006). As the CYN biosynthesis pathway has been recently characterized (Schembri et al., 2001; Mihali et al., 2008), this allowed the development of *C. raciborskii*-specific PCR-based assays, that distinguish between co-occurring CYN-producing and non-CYN producing strains (Rasmussen et al., 2008; Orr et al., 2010; Moreira et al., 2011b; Al-Tebrineh et al., 2012).

*Cylindrospermopsis raciborskii* CYN production is associated with the presence of the *cyr* gene cluster (Sinha et al., 2014), which presents some variation among toxic strains (Jiang et al., 2014). PSP toxins produced by a *C. raciborskii* strain were firstly identified in 1999 in Brazil (Lagos et al., 1999). The most representative and toxic of PSP toxins are saxitoxins (STXs), a class of neurotoxic alkaloid, containing several isoforms with different toxicities (Zingone and Enevoldsen, 2000). Another strain in Brazil was shown to produce predominantly neosaxitoxin (NSTX), with STX as a minor component. Afterward, another Brazilian strain demonstrated to produce at least five STX analogs: STX, gonyautoxin decarbamoylsaxitoxin (dcSTX), NSTX, and a non-described STX analog (Molica et al., 2002). STX and its analogs have been detected in marine organisms, both in dinoflagellates (Harada et al., 1982), filamentous cyanobacteria (Carmichael et al., 1997) and in heterotrophic bacteria (Gallacher and Smith, 1999). In freshwaters, these molecules are mainly associated with cyanobacteria species other that *C. raciborskii* (Kaas and Henriksen, 2000; Pereira et al., 2000). Currently, only Brazilian strains were shown to produce PSP toxins (Figure 1), and their production seems to be related to water hardness (Carneiro et al., 2013). Interestingly, a recent study demonstrated that some Brazilian *C. raciborskii* strains had both *sxt* genes, responsible for the production of STX, and also *cyr* genes fragments, responsible for the production of CYN. Still, the strains only produced STX and not CYN. These results may imply that a modification of toxin production took place along the evolutionary history (Hoff-Risseti et al., 2013). Recently STX was found in a *C. raciborskii*-dominated bloom in Greece, however it was not possible yet to determine if the producer was *C. raciborskii* or *Aphanizomenon flos-aquae* which was also present in the studied area (Gkelis and Zaoutsos, 2014). Molecular methods for the detection of *C. raciborskii* PSP producing strains, analogous to those available for CYN, have only recently begun to be developed, and thus the monitoring of these strains remains more problematic. The detection of both these toxins should use chemical, biochemical or molecular methods or a combination of these (Moreira et al., 2014). Also relevant is the possibility of the production of uncharacterized toxic metabolites by *C. raciborskii*, as it was already demonstrated in some strains isolated from Europe (Poniedziałek et al., 2015), some of which were neurotoxic (Vehovszky et al., 2013) This reinforces the necessity for documenting *C. raciborskii* presence and its spread into new areas.

## Expansion and Phylogeography

**FIGURE 2:**
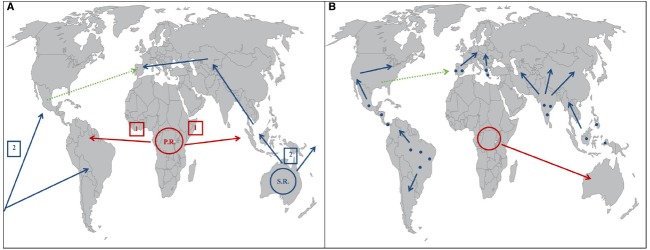
**(A)** Global expansion routes of *C. raciborskii* according to Padisák (1997) with a primary radiation center (P.R.) based in Africa, and a secondary radiation center (S.R.) based in Australia. The green arrow represents posterior migratory movements, according to Cirés et al. (2014). **(B)** Global expansion routes of *C. raciborskii* according to Haande et al. (2008). Blue points represent hypothetical warmer zones of refuge in the Eurasian and American continents, and the blue arrows represent the continental expansion of *C. raciborskii* from those areas. The red arrow represents the expansion of *C. raciborskii* from the African continent to Australia. The green arrow represents posterior migratory movements, according to Cirés et al. (2014).

Different hypotheses have been put forward to explain the origin and spread routes of *C. raciborskii*. The first proposal, defined by Padisák (1997), suggests a primary origin in the tropical lakes of Africa, with subsequent spreading to other equatorial regions like Indonesia and Central America. A secondary radiation center would be situated in Australia and account for the dispersion to tropical, subtropical, and temperate regions. These inferences were based in epidemiological and hydrological data, and on the physiological characteristics of the species. The different climates in the Australian continent would have allowed *C. raciborskii* invasive strains to develop shade and salt tolerance characteristics, important for the expansion into temperate climates. The dispersion to temperate climates from Australia possibly had two routes, one oceanic through the Pacific Ocean toward North and South America, and a continental route that led to Central Asia and then reached Europe (Figure 2A; Padisák, 1997).

Different mechanisms may explain the intercontinental dispersal of this species. Migratory birds can transport akinetes in its feet and guts, while imported tropical fishes may carry the vegetative form of *C. raciborskii*. Unintentional human transport, in recreational boats, commercial ship ballast water, aquaria or transfer of scientific samples may also be significant dispersal vectors (Atkinson, 1972, 1980). Another hypothesis refers to natural viral-like particles that cause *C. raciborskii* lysis (Pollard and Young, 2010). The shorter trichomes that form as a result of cell lysis, would then lead to an increase in the rate of dispersal of *C. raciborskii*. The first approach to characterize the phylogeography of *C. raciborskii* with genetic data was conducted with strains from Australia, Europe, and America (Dyble et al., 2002). The results showed a separation into European, Australian, and American groups, but did not clearly distinguish strains from these last two continents. Work by Neilan et al. (2003) further examined *C. raciborskii* phylogeography. The studied strains showed significant divergence, supporting the existence of distinct Australian, European and North/South American phylotypes (Neilan et al., 2003). Great genetic similarity between European and Australian strains, suggests a shift from the Australasian region to Europe, in agreement with the initial proposal by Padisák (1997).

Gugger et al. (2005) carried out a genetic study of *C. raciborskii* with strains from Africa, America, Australia, and Europe. The results differentiated American, European, African and Australian clusters. Contrary to Padisák (1997) however, the data suggested that the recent invasion of temperate climates by *C. raciborskii* did not result from colonization from Africa and Australia. Rather, it was indicative that extreme climatic conditions like glaciations and dry climate, during the Pleistocene age, led to a general extinction of *Cylindrospermopsis* in the different continents, except in some warmer areas. As a consequence of the recent climate warming, *Cylindrospermopsis* expanded progressively from those areas to more northern zones in Europe and America. This agrees with the observation that optimum values of light and temperature for the European strains are similar to those of the tropical strains. Therefore the European strains could have had their origin in these warmer zones of refuge. In order for this hypothesis to be confirmed, a relationship between the European strains and areas of potential refuge in the Eurasian continent needs to be established.

The work of Haande et al. (2008) aimed at a phylogenetic analysis of *C. raciborskii* strains through analysis of concatenated DNA sequence data, which allowed for more robust results in differentiating strains from different geographic areas. The first approach, divided the samples into American, European and Australian–African groups. The results also revealed a further division into African and Australian groups, which is in disagreement with Dyble et al. (2002). *C. raciborskii* also shows a surprisingly low degree of diversity in comparison with other groups of cyanobacteria (Haande et al., 2008). Haande et al. (2008) proposed the hypothesis of a relatively recent spread of *Cylindrospermopsis* across America and Europe from warmer areas of refuge as put forward by Gugger et al. (2005), together with an also recent colonization of Australia by African strains (Figure 2B).

The first study with strains from all the five continents (Moreira et al., 2011a) was important to investigate the hypothesis of a tropical origin for *C. raciborskii* as suggested by Padisák (1997). In that study, the European strains were found to be most closely related to Asian and Australian strains. The authors suggest that the recent invasion of *C. raciborskii* to Europe may have had its origin from the Asian and/or Australian continents, in accordance with Padisák (1997). A very recent study however, revealed significant results suggesting that the theories of Padisák and Haande cannot explain by themselves the phylogeography and routes of global dispersion of *C. raciborskii*, and therefore alternative theories are needed (Cirés et al., 2014). Based on *cpcBA-IGS* and *nifH* gene analysis data, this study revealed that certain *C. raciborskii* strains from Spain were grouped together with Tunisian strains. Moreover, these strains were almost identical to strains found in America (USA and Mexico), while being distinct from the rest of the European strains. One interpretation for these results assumes the existence of different ecotypes or genotypes in the European continent, like it has been observed in America. One other reading is that the strains of *C. raciborskii* may have migrated across the ocean from America to Europe in a subsequent migration by the transport of akinetes or trichomes (Atkinson, 1972, 1980). According to this last hypothesis, it can be inferred that there may have been more recent migrations in other parts of the world, after the migrations routes proposed by Padisák and Haande (Figures 2A,B). Overall, the high genetic relationship between strains from temperate and tropical regions highlights the success and tolerance of this invasive species. The geographical spread of cyanobacteria can generally be regarded as a dynamic process that enables variation of the dominant genotype of a population (Chonudomkul et al., 2004). The phenotypic and genetic variability of *C. raciborskii* reflects the existence of different ecotypes, therefore its expansion can be explained by the selection of ecotypes with different environmental requirements (Chonudomkul et al., 2004; Piccini et al., 2011). Different proposed global migratory routes are in favor of these assumptions (Padisák, 1997; Gugger et al., 2005; Haande et al., 2008). Nevertheless, recent data suggests that these hypotheses lack some coherence in explaining the global distribution of some strains (Cirés et al., 2014). Therefore, to obtain a clearer relationship between genetic variation and geography of this species, it would be ideal to conduct further experimentation with several new strains, particularly from the Asian and African continents, which have been scarcely examined, as well as from different geographical areas from other continents.

The recent work of Moreira et al. (2015) addressed some of these concerns, using strains from all continents and a concatenated system including three different genetic markers: 16S rRNA gene, 16S–23S rRNA larger fragment (ITS-L), and RNA polymerase *rpoC1*. The results suggest an alternative origin of *C. raciborskii*, centered in the tropical areas of the American continent. From the original center of dispersion in America, the authors suggest a spread of the cyanobacterium to Africa when the two continents were merged, as there would be no possibility of intercontinental spread of this freshwater species. However, the lack of fossil records, make it impossible to determine exactly when this migration occurred. After that migration there was the spread to the Asian and Australian continent and lastly to Europe which happened in a shorter time scale. This assumption is based on the genetic similarity between the European and the Asian and Australian isolates, and on the fact that the American strains are the most diverging suggesting that former geographical strains appeared later in the evolution of this species. These evidences are consistent with the relatively recent description of this species in Europe.

## Ecophysiology

### Temperature

*Cylindrospermopsis raciborskii* strains from different parts of the world exhibit wide tolerance to temperature values (Briand et al., 2004; Vidal and Kruk, 2008; Kokociński et al., 2010; Everson et al., 2011), and are capable of sustaining biomass at temperatures as low as 14–17°C (Chonudomkul et al., 2004; Piccini et al., 2011) or even 11°C (Bonilla et al., 2012). The adaptation to low temperatures in this species may be a consequence of polar carotenoids production (Várkonyi et al., 2002). On the other hand, 35°C was the highest temperature value where net positive growth of *C. raciborskii* was observed (Briand et al., 2004). Modeling data demonstrated that *C. raciborskii* blooms are likely to occur between the temperatures of 25–32°C (Recknagel et al., 2014), with higher temperatures being favorable to the bloom formation, which explains their regular appearance in tropical regions (Soares et al., 2012).

Due to the wide tolerance to temperature values, Bonilla et al. (2012) considered that the classification of *C. raciborskii* as a tropical species, may be a result of lack of information of different geographical strains rather than of any significant limitation of its ecophysiology. It is long thought that the temperature increase is favorable to *C. raciborskii* expansion (Paerl, 1988; Padisák, 1997; Briand et al., 2002; Wiedner et al., 2007; Hadas et al., 2012). The process of akinete germination in particular, is thought to be crucial in the expansion to higher latitudes (Stüken et al., 2006; Wiedner et al., 2007). In colder regions, filaments are present in the water during the stratified ice-free period only, and form numerous akinetes for hibernation (Padisák, 1997, 2003). Akinetes are resistant cells and allow *C. raciborskii* to survive in colder conditions in temperate climates (Fabbro and Duivenvoorden, 1996; Bouvy et al., 1999; Kling, 2009). Although different strains may have different ecotypes, akinete formation by *C. raciborskii* strains seems to be consistently influenced by seasonal rise of temperatures (Mehnert et al., 2013; Yamamoto and Shiah, 2013). *C. raciborskii* is considered to germinate at relatively low temperatures: 22–23°C (Briand et al., 2002), or 17°C (Mischke, 2003). Early warming in spring is particularly relevant, as it may allow this species to germinate earlier, which could constitute a selective advantage over native species, even if the life cycle based on akinetes is the same (Wiedner et al., 2007; Mehnert et al., 2013). A study of the growth requirements of *C. raciborskii* in German lakes, demonstrated that further increase of temperatures would favor the growth of *C. raciborskii* over native species (Mehnert et al., 2010). Sinha et al. (2012) suggested that the increased incidence of *C. raciborskii* in temperate climates is a result of the increase of temperatures in the recent decades. As there is a projected increase of temperature of 5°C by the year 2100, the presence of this species is expected to rise further in temperate areas of the world (Sinha et al., 2012). A mathematical model that simulated *C. raciborskii* life cycle in a lake in northern Germany, confirmed that an increase in water temperatures will probably result in an increase of *C. raciborskii* prevalence (Jöhnk et al., 2011).

Due to the existence of several strains or ecotypes adapted to different temperatures, Chonudomkul et al. (2004) concluded that the expansion of *C. raciborskii* to temperate climates may be the result of selection of clones with different requirements. The work of Wiedner et al. (2007) also suggests a selection of ecotypes, with lower critical temperature for akinete germination, in its northernmost habitats. This assumption is further supported by ecophysiological and genetic data obtained from South American strains (Piccini et al., 2011). Saker and Neilan (2001) demonstrated that temperate strains produce more akinetes than their tropical counterparts, which can illustrate the adaptation of strains to different climates. The interplay between ecology and evolution at the genetic level may accordingly be influential for the spread of this species. Whether the impact of an increase of the global temperature on this species is due to its physiological resilience, or due to the existence of different ecotypes, remains unclear. Nevertheless, global warming will most certainly be determinant in the further spread of this species into temperate climates (Hamilton et al., 2005; Conroy et al., 2007; Sukenik et al., 2012; Paerl, 2014) and *C. raciborskii* is considered one of the examples of freshwater cyanobacterial species with bloom forming potential, favored by global warming conditions (Paerl and Huisman, 2009; Paerl et al., 2011; Carey et al., 2012; Cottingham et al., 2015).

## Light and Water Column Stability

*Cylindrospermopsis raciborskii* demonstrates a high tolerance to light intensities. Growth has been observed over a wide range of values of irradiance, from a few tens to some hundreds of μmol photons m^2^ (Dokulil and Mayer, 1996; Fabbro and Duivenvoorden, 1996; Briand et al., 2004; Berger et al., 2006; Mehnert et al., 2010). Some strains appear to be adapted to particular light conditions, which may explain the occurrence and dominance in some habitats (Pierangelini et al., 2014a). Unlike most cyanobacteria, particularly of tropical origin, this species has tolerance to shade, and benefits from low light intensities (Padisák and Reynolds, 1998; Briand et al., 2002). *C. raciborskii* can form blooms that persist at low light intensities (Padisák, 1997), and this is particularly relevant as blooms are by itself self-shading (Shafik et al., 2001; Briand et al., 2004). The role of buoyancy in this species is also important to optimize light uptake in the water column (Padisák, 1997). Contrary to temperature, solar radiation was not considered a decisive factor limiting *C. raciborskii* expansion into temperate climates (Dokulil and Teubner, 2000; Mischke, 2003). In a subtropical reservoir, Havens (1998) demonstrated that the main factor in the phytoplankton succession was the combined effect of irradiance wind, solar radiation and thermal stability of the water column (Bouvy et al., 1999, 2003). Some physiological studies elucidate partly the adaptation of *C. raciborskii* to a wider range of light intensities, and its influence in photosynthetic activity (O’Brien et al., 2009). *C. raciborskii* cells adapted to high irradiance values showed to be more productive under such conditions, while dark-acclimated cells were more productive in moderate irradiance (O’Brien et al., 2009). Furthermore, carotenoid and phycobilin concentrations, as well as photosynthetic activities, were significantly higher than those of other cyanobacterial species such as *Microcystis* and *Aphanizomenon* (Wu et al., 2009).

The spread of *C. raciborskii* to higher latitudes has been studied regarding both thermal acclimation and photoacclimation (Mehnert et al., 2012). With the increase of light intensities at lower temperatures conditions, *C. raciborskii* is reportedly more susceptible to light stress than the native species *Aphanizomenon gracile* (Mehnert et al., 2012). In these conditions, *C. raciborskii* responds with a greater ratio of photoprotective carotenoids. This may be associated with the distinct biogeographical origins of the two species. *C. raciborskii* is typically dispersed throughout the water column, and is adapted to low light conditions encountered in turbid and eutrophic water (Padisák, 1997; Mehnert et al., 2012; Recknagel et al., 2014). Nevertheless, stratified water column conditions are generally considered to be favorable to this cyanobacterial species (Bouvy et al., 1999, 2003; McGregor and Fabbro, 2000; Berger et al., 2006). A more recent study demonstrated that *C. raciborskii* was dominant in mixed systems, during dry season in Brazil (Soares et al., 2013) and it was also dominant in unstratified regimes with low concentration of dissolved nutrients (Baptista and Nixdorf, 2014). A variation along water depth gradients seems to exist and *C. raciborskii* was dominant in surface waters compared with bottom waters, when in competition with other diazotrophic species (Zheng et al., 2014).

## Nitrogen Dynamics

*Cylindrospermopsis raciborskii* may be considered a generalist in terms of nitrogen (N) usage, due to its facultative diazotrophs. This species can alternate between N_2_ fixation and dissolved inorganic N assimilation, thus responding to environmental variations of this element (Moisander et al., 2012). N_2_ fixation is carried out solely by the terminal heterocyst cells (Plominsky et al., 2013), and allows this species to use low dissolved N systems (Harris and Baxter, 1996; Présing et al., 1996; Padisák and Istvánovics, 1997; Komárková et al., 1999; Dokulil and Teubner, 2000; McGregor and Fabbro, 2000). This ability can also contribute for this species dominance in lakes and reservoirs, particularly over non-N_2_ fixing species, giving it an ecological advantage (Harris and Baxter, 1996; Hadas et al., 2012). Laboratory studies have demonstrated that *C. raciborskii* has different preferences for N sources. Growth rates were fastest with ammonia, then nitrate and lastly urea (Hawkins et al., 2001; Saker and Neilan, 2001; Sprober et al., 2003). In addition, this invading species was shown to grow faster than cosmopolite *Planktothrix* at high ammonia concentrations, suggesting that it can compete with native species from temperate climates (Ammar et al., 2014). N_2_ is less efficiently used than nitrate for cellular assimilation (Shafik et al., 2001), probably because of the cost associated with the establishment of the heterocyst (Turpin et al., 1985). In reservoirs with high concentrations of nitrate, *C. raciborskii* had fewer heterocysts, indicative of its preference for nitrate as N source (Bouvy et al., 1999; Briand et al., 2002). However in tropical reservoirs, conditions of dissolved inorganic N were considered the main factor triggering the formation of *C. raciborskii* (Figueredo et al., 2013).

## Phosphorus Dynamics

*Cylindrospermopsis raciborskii* may be considered an opportunistic species regarding the usage of dissolved inorganic phosphorus (DIP). This species has both a high uptake affinity (Wu et al., 2009) and high storage capacity for phosphorus (P; Istvánovics et al., 2000). These characteristics are beneficial when there are fluctuations of P concentrations (Istvánovics et al., 2000), or vertical gradients of nutrients. In these conditions *C. raciborskii* may use its buoyancy regulation capacity (Fabbro and Duivenvoorden, 1996; Istvánovics et al., 2000). Pulsed additions of DIP were considered favorable to *C. raciborskii* growth, due to its high P storage ability, and are beneficial over DIP constant inputs (Posselt et al., 2009). This capacity may also be important for the germination of akinetes (Padisák and Istvánovics, 1997). The effective uptake and transformation of P was shown to be higher in *C. raciborskii* than in other cyanobacterial species like *Microcystis aeruginosa* and *Aphanizomenon flos-aquae* (Wu et al., 2009). *C. raciborskii* seems able to regulate its physiological metabolism and adapt to low ambient DIP concentrations, by an increase of alkaline phosphatase (ALP), which is considered a defensive mechanism to overcome P limitation (Wu et al., 2011). Furthermore *C. raciborskii* is capable of using of different organic P sources to support its growth, when there is environmental limitation of this nutrient (Bai et al., 2014).

A recent study demonstrated that for an *Aphanizomenon* strain, situations of DIP deprivation caused both an induction of CYN production as well as an upregulation of DIP uptake machinery (Bar-Yosef et al., 2010). CYN causes the stimulation of APase activity in other organisms and *Aphanizomenon* can compete for the released DIP. It is currently unknown how distributed this mechanism is, but several *C. raciborskii* strains are CYN producers and may eventually possess and benefit from this strategy. In conditions of high P concentrations, however, the higher P uptake rate of *C. raciborskii* offers no advantage, and, in this case, a deciding factor in competition between species would be based on different uptake rates of ammonium (Borics et al., 2000). Different nutrient uptake strategies should however be considered collectively to fully explain their role in the growth of *C. raciborskii* populations. For example, it was recently shown that, in a eutrophic area, *C. raciborskii* dominates when the N:P ratio is either very high or very low (Chislock et al., 2014) A synergistic effect of global warming with local nutrient conditions, may better explain the successful invasion of *C. raciborskii* into temperate waters (Sukenik et al., 2012).

## Salinity, pH, and CO_2_

It was recognized that *C. raciborskii* has preference for low salinity conditions, with optimal growth in fresh to oligohaline conditions (Chapman and Schelske, 1997; Padisák, 1997). Elevated salinity values are considered limiting for *C. raciborskii* growth (Moisander et al., 2012). The species is capable of growth in slightly brackish waters, particularly if there are elevated concentrations of dissolved minerals (Briand et al., 2002), or nutrient enrichment conditions (Calandrino and Paerl, 2011). Consequently *C. raciborskii* is capable of invading eutrophying systems of moderate salinity (Calandrino and Paerl, 2011). The climate change conditions that cause alterations to rainfall may modify the salinity conditions of estuarine systems and contribute to the invasiveness of *C. raciborskii* in those ecosystems. Consequently, salinity changes may affect the community as well as have potential impacts on toxin production by *C. raciborskii*.

Regarding pH, *C. raciborskii* has preference for high pH values and was shown to grow between values of 8.1 and 9.4 (Bouvy et al., 1999). More recently, Bonilla et al. (2012) determined the pH values of lakes where there was *C. raciborskii* growth, to be between 5.49 and 9.91, with a median value of 8.2. Increases in the atmospheric partial pressure of CO_2_ will lower pH (Holland et al., 2011). The CO_2_ partial pressure of the atmosphere is expected to increase threefold at the end of the century. This is expected to have consequences on a range of phytoplankton-related physiological processes including photosynthesis (Paerl and Huisman, 2009). Increase in the values of CO_2_, will lower the values of pH and diminish the proportion of CO_2_ to HCO_3_, consequently higher CO_2_ concentrations in the future may lead to competitive disadvantage to *C. raciborskii* (Holland et al., 2011). However, the influence of CO_2_ in the spread of *C. raciborskii* may be negligible (Sinha et al., 2012) when compared to the expected effects of global rising temperatures. Moreover, since *C. raciborskii* is capable of growing with high CO_2_ concentrations, increase of CO_2_ will not likely have a significant effect on the ecological performance of this species (Pierangelini et al., 2014b).

## Allelopathy

Allelopathic activity has been recently suggested to contribute to the geographical expansion of *C. raciborskii*. This hypothesis was first put forward in a study conducted in a southeastern Brazil lake, where this species had become dominant (Figueredo et al., 2007). Exudates from different *C. raciborskii* strains resulted in strong inhibitory effects on the photosynthetic activities of different algal species. Allelopathy was consequently considered by the authors to be an advantageous mechanism in that environment, and also a potentially significant factor in the spread of this species into water bodies in temperate climates (Figueredo et al., 2007). Allelopathy is thought to have a role in the phytoplankton structuring and succession in lakes (Fistarol et al., 2003), and is considered to be relevant in ecological interactions in aquatic habitats (Gross, 2003; Legrand et al., 2003; Leflaive and Ten-Hage, 2007; Leão et al., 2009a). Eventual benefits of allelopathic activity to invasive species may be explained by the fact that the native species lack long periods of coexistence with invasive species, and have not evolved to endure the allelochemicals produced by invasive species (Fitter, 2003). Leão et al. (2009b) tested the allelopathic potential of several *C. raciborskii* strains from Portuguese reservoirs. One strain, LEGE 99043 (formerly strain 4799) significantly inhibited the growth of the ubiquitous microalgae *Ankistrodesmus falcatus*. A subsequent study demonstrated that the allelopathic activity of that strain was influenced by different environmental parameters (Antunes et al., 2012). Phosphorus deprivation, as well higher temperature and light intensities, resulted in higher allelopathic activity. The results suggest that allelopathy may have ecophysiological relevance for this species. Increase of allelopathic activity in high temperature conditions, in particular, may be significant if combined with the role of the climate warming in the spread of *C. raciborskii* into temperate climates (Antunes et al., 2012).

Allelopathy may also have a role on *C. raciborskii* dominance (Mello et al., 2012). This was analyzed in samples from a tropical reservoir, where populations of *C. raciborskii* and *M. aeruginosa* coexisted naturally (Dantas et al., 2008; Soares et al., 2009a). Growth inhibition of *M. aeruginosa* was observed when it was exposed to exudates from mixed cultures with high proportion of *C. raciborskii*, and *Microcystis* colonies were induced in a tested strain of *M. aeruginosa* found growing with *C. raciborskii* (Mello et al., 2012). These results demonstrate that allelopathy may also be important in explaining seasonal dynamics of this species. On the other hand, *C. raciborskii* showed the ability to outgrow *M. aeruginosa* in situations of co-culturing through the production of still uncharacterized allelopathic compounds which mimic CYN action (Rzymski et al., 2014).

## Biotic Interactions

Invasive species like *C. raciborskii* severely influence the invaded communities and species diversity, as they may alter the species dominance structure, nutrient dynamics, and levels of primary productivity (Vitousek, 1990; Mack et al., 2000). Aquatic ecosystems are particularly sensitive to these changes especially if associated with the formation of cyanobacterial blooms. Preliminary studies revealed that *C. raciborskii* is a species with particular resistance to predation, and is generally unsuitable food for zooplankton species (Nogueira et al., 2004; Panosso and Lürling, 2010). The impact of *C. raciborskii* on grazers was studied mostly on Daphnids (Nogueira et al., 2004; Ferrão-Filho et al., 2008; Bednarska et al., 2014) and on rotifers (Soares et al., 2010), resulting in harmful effects to the tested species and consequently negative consequences to their populations.

Strain specificity and toxicity were found to be determinant in the grazing of *C. raciborskii*, and it has been suggested that presence of toxic *C. raciborskii* strains may be a consequence of grazing pressure (Ka et al., 2006). In a lake where the adverse grazing conditions are not present, toxic clones are not selected, and their proportion remains relatively low (Ka et al., 2006). Sensitivity of cladocerans to *C. raciborskii* differs from STX producing and non-STX producing strains and the metabolites produced (Costa et al., 2013). However, this varies among species and some cladocerans can use STX producing *C. raciborskii* as complementary resource with no negative effect to their fitness (Ferrão-Filho et al., 2014). Experimental evidence suggests that CYN producing strains have deep impacts in different groups of organisms. Preliminary studies demonstrated the accumulation of CYN from toxic *C. raciborskii* strains and the contamination of the redclaw crayfish *Cherax quadricarinatus* (Saker and Eaglesham, 1999), the freshwater mussel *Anodonta cygnea* (Saker et al., 2004), tadpoles of the cane toad *Bufo marinus* (Kinnear et al., 2007; White et al., 2007) and zebrafish embryos (Berry et al., 2009) or *Danio rerio* embryos (Ács et al., 2013). This effect is not restricted to toxicity, as non-toxic strains were able to influence the behavior and physiology of *Daphnia magna* (Dao et al., 2013). Overall, the increase of *C. raciborskii* dominance may cause a decrease in the species diversity and richness. Leonard and Paerl (2005) demonstrated that at low *C. raciborskii* densities, zooplankton was more diverse and comprised larger species. At higher concentrations of *C. raciborskii*, the presence of rotifers increased, and the number of microzooplankton species was higher. Consequently high concentration of *C. raciborskii* may shift the zooplankton community structure toward smaller species (Leonard and Paerl, 2005).

*Cylindrospermopsis raciborskii* bloom phase and its toxicity may have different effects in the local community biodiversity responding to *C. raciborskii* bloom characteristics (Moustaka-Gouni et al., 2006; Burford et al., 2014). Conversely, field studies demonstrated that *C. raciborskii* dominance may lead to an increase of Shannon–Wiener diversity of phytoplankton (Kokociński et al., 2010), zooplankton species (Bouvy et al., 2000; Soares et al., 2009b), or cause advantages for micro-grazers species (Davis et al., 2012). The presence and growth of *C. raciborskii* itself may be dependent on other groups, copepods for instance may conduct preferential grazing of other species of algae allowing for *C. raciborskii* expansion (Hong et al., 2013). Likewise, it was suggested that the phytoplankton-associated bacterial communities may also result in the dominance of this species (Bagatini et al., 2014). Competition with other cyanobacterial species also occurs, and the dominance of this species against *M. aeruginosa* was found to be dependent of the specificity of strains and environmental conditions (Marinho et al., 2013). The current invasion and domination of *C. raciborskii* in temperate waters may cause a displacement of native phytoplankton species, either due to its toxicity or to the impacts caused to the food chain (Sukenik et al., 2012). Overall, the disparity of reported effects of *C. raciborskii* on different biota and ecosystems may be a result of this species high plasticity (Kokociński et al., 2010) and suggests distinct effects on different trophic levels.

## Conclusion

Due to the presence of *C. raciborskii* in geographical zones with distinct climates, as well as its multi-continent occurrence, the classification of this species as cosmopolitan seems adequate. The phylogeography of this species was determined by different studies, for which a tropical primary radiation center is consistent. The scenario of an expansion from warmer zones of refuge, from a secondary radiation center in the Australian continent may only be confirmed when more strains are analyzed. A significant reevaluation of these theories may also be needed according to some more recent phylogeographic studies. The most comprehensive of the studies so far, which comprised strains from all continents with several genetic markers, propose an origin of this species in the tropical areas of the American continent. It is still not entirely possible to determine if the spread of *C. raciborskii* into different climates is a result of great physiological plasticity of the whole species or of the existence of ecotypes with different environmental requirements. It is nevertheless evident that this species shows flexible nutrient dynamics, which are significant to its invasive characteristics. The spread of *C. raciborskii* seems to be associated to global warming conditions, and to allelopathy. Still, the ecological impact of *C. raciborskii* in the invaded communities is not completely clarified in terms of its magnitude and consequences. The occurrence of *C. raciborskii* toxic strains is likely to remain underestimated, and further reports of toxic strains in other geographic regions are to be expected. Both the ecophysiological features of *C. raciborskii* and climate change conditions, should lead to an overall increase of *C. raciborskii* reports. This is of particular relevance due to the potential of toxic bloom formation by this species.

### Conflict of Interest Statement

The authors declare that the research was conducted in the absence of any commercial or financial relationships that could be construed as a potential conflict of interest.
